# 

**Published:** 1911-06-18

**Authors:** 


### HOSPITALS AND THEIR SPECIAL NEEDS.

**Cancer Hospital (Free), Fulham Road, S.W.**—Funds needed for general expenses and for building and equipping a Cancer Research Department.

**Central London Ophthalmic Hospital, Gray's Inn Road, W.C.**—£8,000 is still required to complete necessary rebuilding, and new annual subscriptions towards maintenance.

**Chelsea Hospital for Women, Fulham Road, S.W.** New annual subscriptions required to meet deficiency in income.

**City of London Lying-in Hospital, City Road, E.C.**—Greatly needs help to pay off the heavy debt entailed through compulsory rebuilding.

**Evelina Hospital for Children, Southwark Bridge Road, S.E.**—£8,000 must be raised annually to defray the cost of maintaining this hospital, which is situated in one of the poorest districts of the Metropolis. Additional support is urgently needed in the form of new annual subscriptions.

**Great Northern Central Hospital, Holloway Road, N.**—Financial position causing grave anxiety to committee. £18,000 required annually, of which £6,000 only is assured.

**Guy's Hospital, London Bridge, S.E.**—£60,000 required to provide children's wards and badly needed increase of beds for special departments.

**Hampstead General Hospital and North-West London Hospital** (Amalgamated).—£5,000 is required to enable the new out-patients' department to be opened free of debt. Half the amount has already been promised if the balance is raised by the end of the year.

**Hospital for Consumption, Brompton, S.W.**—£30,000 required annually towards the maintenance of the hospital, sanatorium, and convalescent home on the Chobham Ridges. As the fixed income is under £3,000, the committee earnestly appeal for additional support.

**Hospital for Sick Children, Great Ormond Street, W.C.**—New subscriptions urgently required to enable this hospital to cope with increased work.

**King's College Hospital, Lincoln's Inn Fields, W.C.**—An additional £6,000 is needed annually for the maintenance of the hospital and £100,000 toward the removal fund.

**London Homœopathic Hospital, Great Ormond Street, W.C.**—An appeal is made for £1,500 to furnish the new wing now completed.

**National Hospital for Paralysed and Epileptic, Queen Square, W.C.**—Special appeal is made for new annual subscriptions.

### HOSPITALS AND THEIR SPECIAL NEEDS.

**Metropolitan Hospital, Kingsland Road, N.E.**—£2,500 is still required to meet the cost of special repairs and improvements imperatively needed. £100 has already been promised, provided that a further £900 is collected by the end of the year. The committee very earnestly appeal for assistance.

**Paddington Green Children's Hospital, W.**—An earnest appeal is made for funds towards the maintenance of the hospital, which is entirely dependent on voluntary contributions for support.

**Prince of Wales's General Hospital, Tottenham, N.**—Special appeal is made for new annual subscriptions, also for donations towards the liquidation of an old accumulated debt of £4,000.

**Queen Charlotte's Lying-in Hospital, Marylebone Road, N.W.**—£2,000 still required to pay for the enlargement of Nurses' Home, and new annual subscriptions are needed to meet deficiency in ordinary income.

**Queen's Hospital for Children (late "North-Eastern" Hospital), Hackney Road.**—Donations and new annual subscriptions required towards maintenance and to pay off overdraft on bankers of £3,000. The hospital has an endowed annual income of £340 only, whilst the expenditure is over £13,000.

**Royal London Ophthalmic Hospital, City Road, E.C.**—Urgent appeal is made for donations and subscriptions. Adequate support cannot be obtained.

**Royal Free Hospital, Gray's Inn Road, W.C.**—As the necessary annual expenditure exceeds the reliable income by no less than £10,000, an appeal is made for new annual subscriptions.

**Royal Hospital for Incurables, Putney Heath (Office, 4 St. Paul's Churchyard, E.C.).**—Costs £35,000 a year to maintain, of which only £6,000 can be relied upon.

**St. George's Hospital, Hyde Park Corner, S.W.**—Deficit of £15,759. Additional subscriptions urgently needed toward maintenance.

**St. John's Hospital for Diseases of the Skin, Leicester Square, W.C.**—A special appeal is made for donations toward the rebuilding of the in-patients' department.

**St. Mary's Hospital, Paddington, W.**—To maintain the hospital during 1911 a sum of £15,000 is still required. Appeal is earnestly made for assistance.

**Samaritan Free Hospital, Marylebone Road, N.W.** Funds greatly needed to meet heavy current expenses and for necessary structural improvements, which must be carried out without delay.

**Seamen's Hospital Society, Greenwich, S.E.**—An urgent appeal is made for increased support. Over 30,000 patients were treated last year in the hospital and its branches.

### OTHER INSTITUTIONS AND THEIR SPECIAL NEEDS.

**Church Army, 55 Bryanston Street, Marble Arch, W.**—Donations urgently needed to help the unemployed and outcast. A scheme for emigrating lads to Australia has recently been inaugurated.

**London Orphan Asylum, Watford (Office, 3 Crosby Square, E.C.).**—£15,000 has to be raised annually from voluntary sources to enable the work of this charity to be carried on untrammelled by debt.

**Metropolitan Convalescent Institution, 32 Sackville Street, W.**—About £14,000 a year is required to maintain the four Homes.

**Mary Wardell Convalescent Home, Stanmore, Middlesex.**—An effort is now being made to raise an Endowment Fund and an appeal is made for donations towards this object.

**National Children's Home and Orphanage, Bonner Road, N.E.**—£50,000 urgently required for extension purposes which are necessitated by the increasing demand upon the accommodation provided by the orphanages and its branches.

